# Skin Autofluorescence and Mortality in Patients on Peritoneal Dialysis

**DOI:** 10.1097/MD.0000000000001933

**Published:** 2015-11-13

**Authors:** Emília Mácsai, Attila Benke, István Kiss

**Affiliations:** From the 3rd Dialysis Centre of B. Braun Avitum Hungary CPLC, Veszprém (EM, AB); and 1st Dialysis Centre of B. Braun Avitum Hungary CPLC, Department of Nephrology-Hypertension and Geriatrics, St Imre University Teaching Hospital and Div. Sect. of Geriatrics, 2nd Department of Internal Medicine, Semmelweis University, Budapest, Hungary (IK).

## Abstract

Skin autofluorescence (SAF) is a proven prognostic factor of mortality in hemodialysis patients. Traditional and nontraditional risk factors are almost equivalent in peritoneal dialysis (PD), and cardiovascular disease (CVD) is the leading cause of death. Moreover, peritoneal glucose absorption accelerates the degenerative processes of connective tissues as in diabetes. In our study, we examined the predictive value of SAF for total mortality in the PD population.

Data were collected from 198 prevalently adult Caucasian PD patients. One hundred twenty-six patients (mean age 66.2 y, men [n = 73], diabetes ratio 75/126) had anamnestic CVD (coronary heart disease, cerebrovascular disease, peripheral arterial disease). Initially, we evaluated factors affecting SAF and CVD by multivariate linear regression. Survival rates were estimated by recording clinical and demographic data associated with mortality during a 36-month follow-up using the Kaplan–Meier method. Analyses were further stratified based on the presence or absence of CVD and SAF levels above or below the upper tercile 3.61 arbitrary units.

Skin autofluorescence was influenced by CVD (*P* < 0.01, 95% confidence interval [CI] 0.1–0.5) and white blood cell counts (*P* < 0.001, 95% CI 0.031–0.117). According to the Spearman correlation, SAF correlated with peritoneal cumulative glucose exposure (*P* *=* 0.02) and elapsed time in PD (*P* = 0.008). CVD correlated with age (*P* < 0.001, 95% CI 1.24–1.65) and diabetes (*P* < 0.001, 95% CI 2.58–10.66). More deaths were observed in the high SAF group than in the low SAF group (34/68 vs 44/130; *P* = 0.04). Comparing the CVD(−) low SAF group survival (mean 33.9 mos, standard error [SE] 1.39) to CVD(+) low SAF (mean 30.5 mos, SE 1.37, *P* = 0.03) and to CVD(+) high SAF group (mean 27.1 mos, SE 1.83, *P* = 0.001), the difference was significant.

In conclusion, among PD patients, SAF values over 3.61 arbitrary units seem to be a predictor of mortality. The relationship among peritoneal glucose exposure, CVD, and diabetes suggests its suitability to characterize systemic cumulative glucose load in this patient population.

## INTRODUCTION

Incidence of mortality in patients on renal replacement therapy remains unacceptably high, and is affected not only by pre-existing medical conditions but also by dialysis modalities and kidney failure.^1^ Over the past few decades, there has been an increase in the average age of patients requiring dialysis, multiple morbidities due to diabetes have become more widespread, and survival disadvantages to the general population have become more and more pronounced.^2^ Patients with diabetes as a primary renal disease have higher mortality rates than those with diabetes as a comorbid condition, indicating that diabetes-induced organ damage probably affects survival.^3^ Traditional cardiovascular risk factors, including elevated serum cholesterol levels, blood pressure, smoking, obesity, and diabetes, contribute to mortality in dialyzed patients.^4^

Hemodialysis and peritoneal dialysis (PD) display a slightly different pattern of mortality predictors: residual renal function, diabetes, age, malnutrition, inflammation, and protein loss.^5^ Advanced-stage chronic kidney disease (CKD) is associated with complex physiological and metabolic disturbances that are linked to poor overall and cardiovascular outcomes. Combined effects of specific hormonal, inflammatory, nutritional, and metabolic factors may play key roles in mortality.^6^ The survival of diabetic PD patients is worse than that of the patients in the nondiabetic group, which can be mitigated by diet control, optimal PD-solution use, and meticulous predialytic control of cardiovascular risk factors.^7^ Diabetes has been found to predict all-cause death in patients with high-sensitive C-reactive protein (hsCRP) >3 mg/L or serum albumin <38 g/L levels, although the outcome of patients with negative inflammatory status was the same as that of nondiabetic individuals. Therefore, evaluation and correction of inflammation in PD might improve survival even in diabetic patients.^8^

In hemodialysis patients, skin autofluorescence (SAF) has been found to be an independent predictor of mortality and to strongly correlate with CVD,^9^ although in a later study, SAF showed no correlation with cardiovascular mortality.^10^Another study showed that SAF was significantly associated with cardiovascular mortality of patients on chronic hemodialysis.^11^ The association of SAF with the degree of atherosclerosis in a multivariate analysis proved to be independent of age, sex, diabetes, or renal failure.^12^

Molecular details of generation and accumulation of advanced glycation end products (AGEs) have been linked to increased risk of macrovascular and microvascular diabetic complications.^13^ Moreover, AGEs are considered a subgroup of uremic toxins, because the accumulation of AGEs seems to be influenced by a decline in renal function. Moreover, SAF is a strong marker of cardiovascular mortality in CKD patients.^14^

Dialysis is moderately effective for the clearance of AGE fragments. Unfortunately, several types of renal replacement therapy contribute to AGE formation by increasing oxidative stress.^15^ In end-stage renal disease, SAF seems to be involved in arterial stiffness,^16^ diastolic dysfunction,^17^ dialysis-related spondylosis, and arthropathy,^18^ and is not altered by a single hemodialysis treatment alone.^19^ Indeed, SAF and aortic stiffness were higher in PD patients than in HD patients even after adjustments for baseline characteristics.^20^ In both dialysis modalities, there was a positive association between SAF and aortic stiffness. In the PD population, SAF correlated with kidney disease duration and dialysis vintage; the anuric, diabetic, and older patients had higher SAF levels.^21^ In PD patients, SAF correlated with the duration of PD and glucose exposure dose and independently associated with cardiovascular morbidity. Multivariate analysis revealed that glucose exposure dose and SAF were the main risk factors for cardiovascular morbidity after adjustment by age, sex, and other traditional or uremic-related risk factors.^22^

An optimal glycemic marker in PD is currently unavailable. According to the results of the continuous glucose monitoring system (CGMS), in cases of high transport status, the maximum glucose value and its increment after peritoneal exchange seemed to be more intensive in the subgroup of diabetic patients. The mean 24-hour subcutaneous glucose concentration of nondiabetic patients was also unexpectedly elevated. The percentage of glucose levels >5 mmol/L was significantly influenced by a higher glucose concentration in PD fluids and a higher peritoneal transport status.^23^ Even in integrated assessments over 3 to 4 years, hemoglobin A1C (HbA1C) weakly correlated with the change in tissue skin AGE levels, suggesting that longer glucose exposure had an effect on connective tissue structure alterations.^24^ In the case of patients with type II diabetes, SAF also was predictive of neuropathy and albuminuria.^25^ Also with regards to age and diabetes, the switch to icodextrin-based PD solution was also found to be a factor, and is likely associated with increased AGE exposure in patients on PD as a consequence of factors necessitating the regime change.^26^

Therefore, we decided to assess the impact of SAF values on patients’ mortality rate in a PD population, together with a consideration of other predictors of mortality.

## METHODS

### Setting and Participants

This clinical study included 198 patients who were on PD treatment at 10 regional dialysis centers of the B. Braun Avitum Hungary CPLC Dialysis Network in spring 2010. A total of 128 patients were regularly treated with conventional lactate-buffered glucose-based PD solutions (Dianeal, Baxter Company Deerfield, IL). The remaining 70 patients had been previously switched to a dialysis regime, including 1 glucose-free icodextrin solution (mainly night time) per day. In these patients, the long dwell PD solution had been replaced by icodextrin for about 13.5 months (median, interquartile range [IQR] 7–23 mos) before SAF assessment. The single-chamber heat sterilization PD solutions were manufactured, stored, and transported according to the current provider standards.

Patients were treated in accordance with relevant national guidelines,^27^ which are, in essence, identical to the current International Society for Peritoneal Dialysis (ISPD) guidelines.^28^ There were 3 patients (at least 1 y) after renal transplantation. All patients were considered Caucasian with Fitzpatrick classification skin types I to IV (ie, had a light skin). The minority of patients (27/198) were on automated peritoneal dialysis (APD). Exclusion criteria in this study included any history of malignancy, presence of local skin disorders, jaundice, and peritonitis within a month. The patients were followed up for the next 36 months, and deaths and causes of loss were recorded. Among the patients, 126 had overt CVD: 114 ischemic cardiac disease, 63 peripheral arterial insufficiency, and 32 cerebrovascular abnormalities.

### Quantitative Variables

#### Skin Autofluorescence Measurements

The Reader device used (DiagnOptics, Groningen, the Netherlands) for measurements also applied a new algorithm for the adjustment of SAF values according to skin color. The measurements between March and May 2010 were performed at room temperature during PD visits according to the manufacturer's instructions.^29^ SAF values were expressed in arbitrary units (AU) calculated by dividing the average light intensity emitted by skin (per nm over the range from 420 nm to 600 nm) by the average excitation light intensity emitted by internal light source of the device (per nm over the range from 300 nm to 420 nm), and the quotient was multiplied by 100.

### Outcome Variables

#### Follow-up and Total Mortality

Each PD patient's cumulative exposure to PD glucose was calculated using data from strict daily follow-up clinical records, multiplying the daily exposure with the number of days on a given regime. Medical history regarding cardiovascular disorders and self-reported smoking habits was also obtained. Our observational study was approved by the County Hospital Ethical Committee (permission number: K-1411–2/2010) and patients gave their written informed consent. After 36 months, data were taken from the dialysis units electronic registers and evaluated for survival.

### Statistical Analysis

Parameters following normal distribution were reported using mean and standard deviation. Parameters with non-normal distribution, such as C-reactive protein (CRP), parathyroid hormone (PTH), glutamic oxaloacetic transaminase (GOT), glutamic pyruvic transaminase (GPT), alanine aminotransferase (ALP), and gamma-glutamyltransferase (GGT), were reported using median and lower–upper quartile values (Q1–Q3). Differences between the two independent groups were compared using Student *t* test and Mann–Whitney *U* test. Univariate and stepwise multivariate linear regression analyses were also performed to determine the factors influencing SAF. Logistic regression was applied to determine factors influencing CVD. Kaplan–Meier analysis and log-rank test were used to evaluate survival results. The statistical analysis was performed using the STATISTICA software package version 10 (Tulsa, Oklahoma). We considered *P* = 0.05 as significant.

## RESULTS

### General Characteristics and Laboratory Data

In total, 237 patients were screened, and 198 patients were enrolled and completed the trial. Table 1 and Table 2 show the baseline characteristics of the study sample population. The mean age of the patients with CVD (CVD[+]) was 66.2 years, whereas the mean age was only 55.8 years in the group without CVD (CVD[−]). The demographics were significantly different between the two groups, including body mass index (BMI), percent of diabetes mellitus, neuropathy, retinopathy, SAF, diastolic blood pressure, total cholesterol levels, fasting glucose level, PTH, and serum calcium × phosphate (CaxP).

**TABLE 1:**
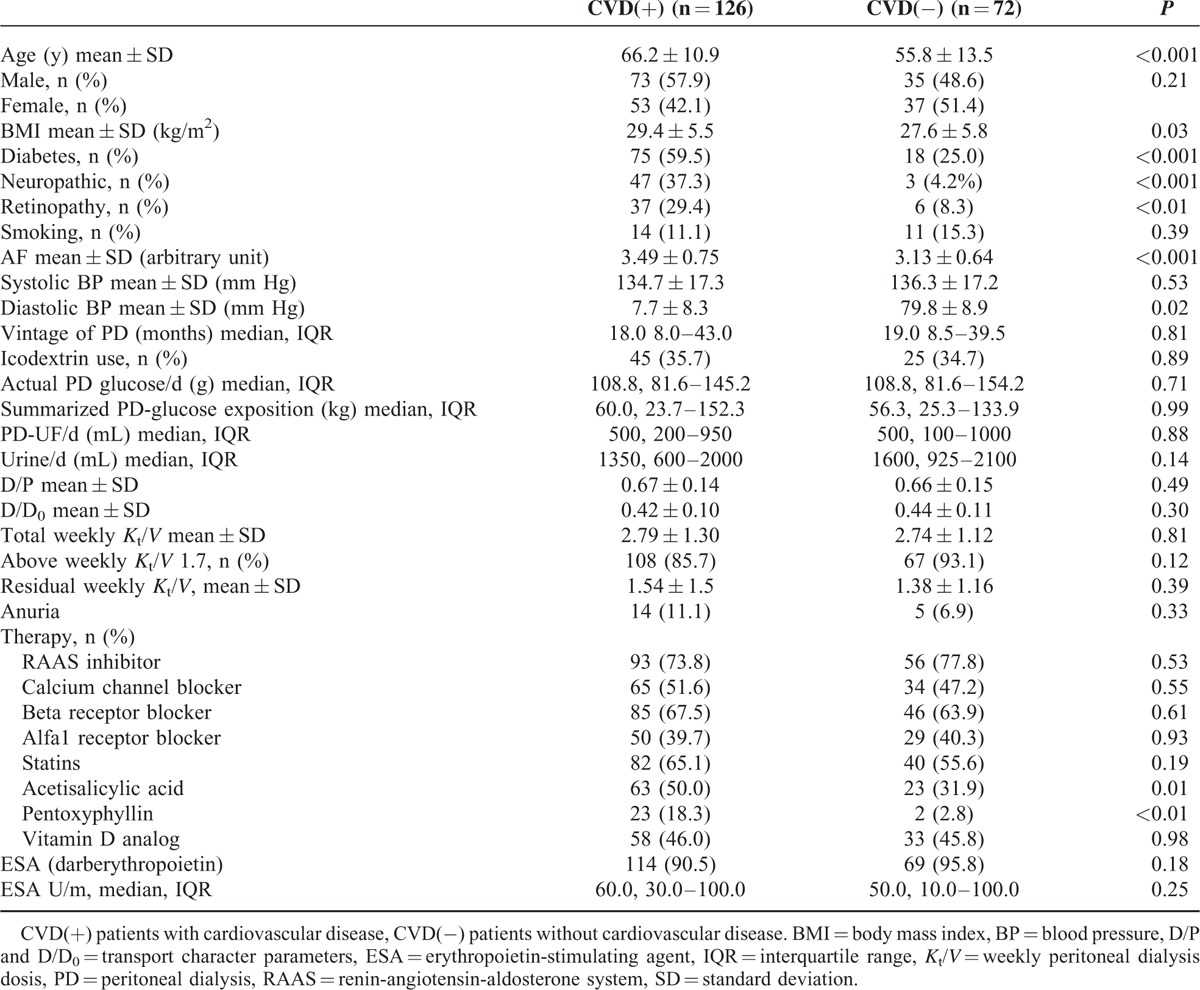
Demographic and Clinical Data of Patients on Peritoneal Dialysis With or Without Cardiovascular Disease

**TABLE 2:**
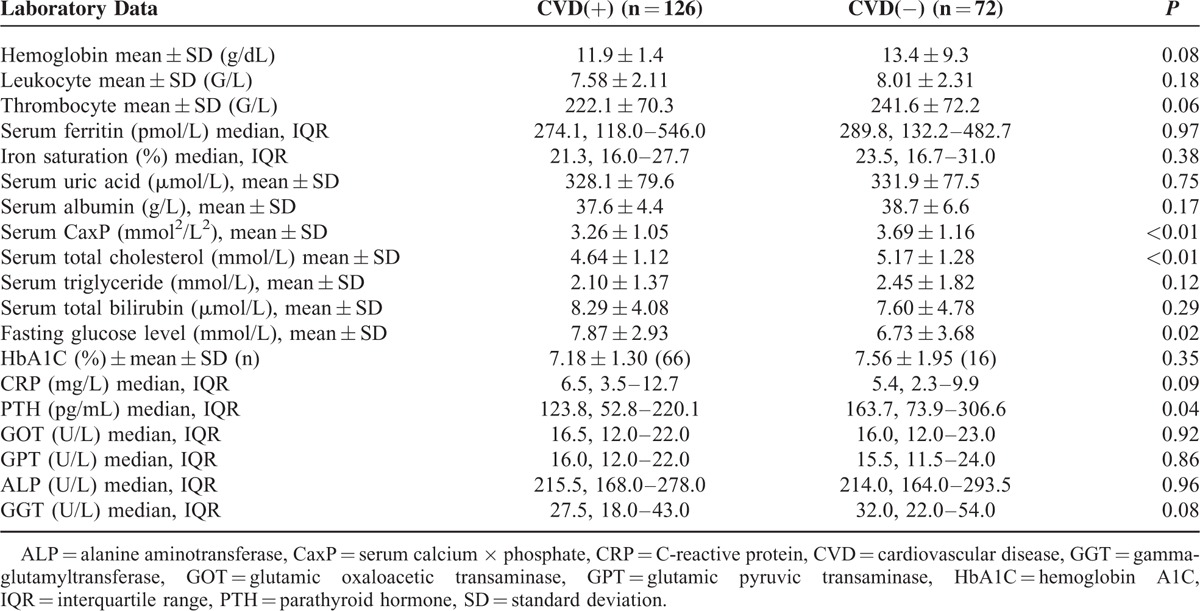
Laboratory Data of Patients on Peritoneal Dialysis With or Without Cardiovascular Disease

### Determinants of Initial Skin Autofluorescence

Univariate linear regression analysis revealed that patients’ age, presence of CVD, time on PD, icodextrin use, ultrafiltration, leukocyte count, diastolic blood pressure, serum albumin level, serum total cholesterol level, serum GOT, and diabetes had a significant impact on patients’ SAF values. Though stepwise multivariate evaluation indicated (Table 3) a significant effect from known CVD (*P* < 0.01, 95% confidence interval [CI] 0.10–0.50), icodextrin use (*P* < 0.01, 95% CI 0.11–0.51), leukocyte count (*P* < 0.001, 95% CI 0.031–0.117), diastolic blood pressure (*P* = 0.03, 95% CI −0.024 to −0.001), and serum total cholesterol (*P* = 0.03, 95% CI −0.175 to −0.01).

**TABLE 3:**
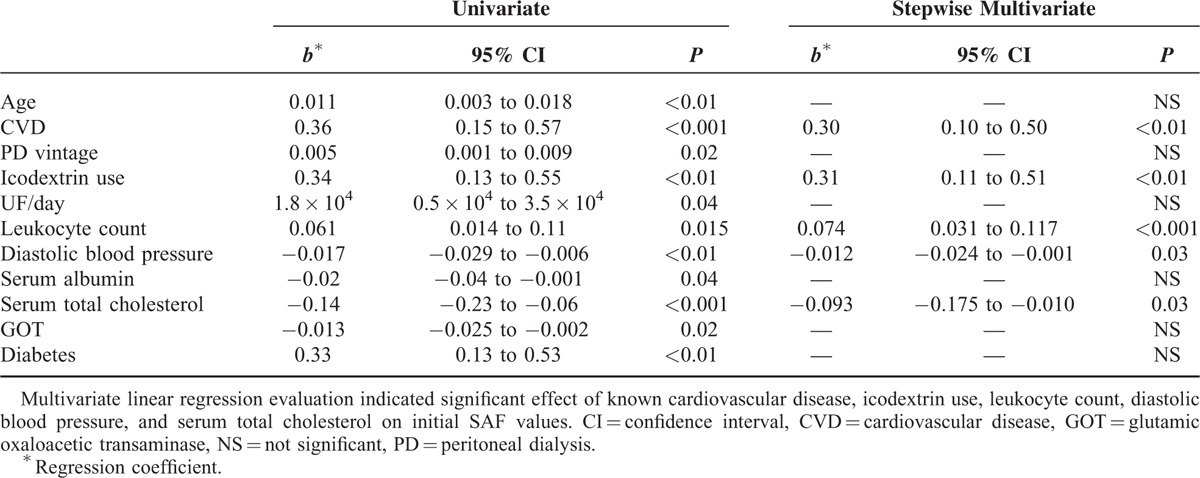
Multivariate Linear Regression of Factors Effecting Initial SAF Values

### Cardiovascular Disease in the Study Cohort

According to stepwise multivariate logistic regression results (Table 4), the presence of CVD was strongly correlated with age and diabetes.

**TABLE 4:**
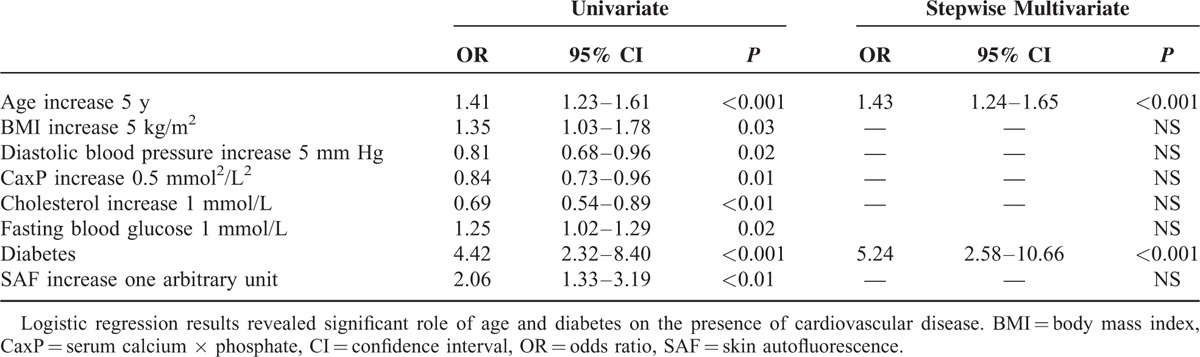
Logistic Regression Results of Clinical Factors on the Presence of Initial Cardiovascular Disease

### Survival Results in Peritoneal Dialysis Patients

**FIGURE 1:**
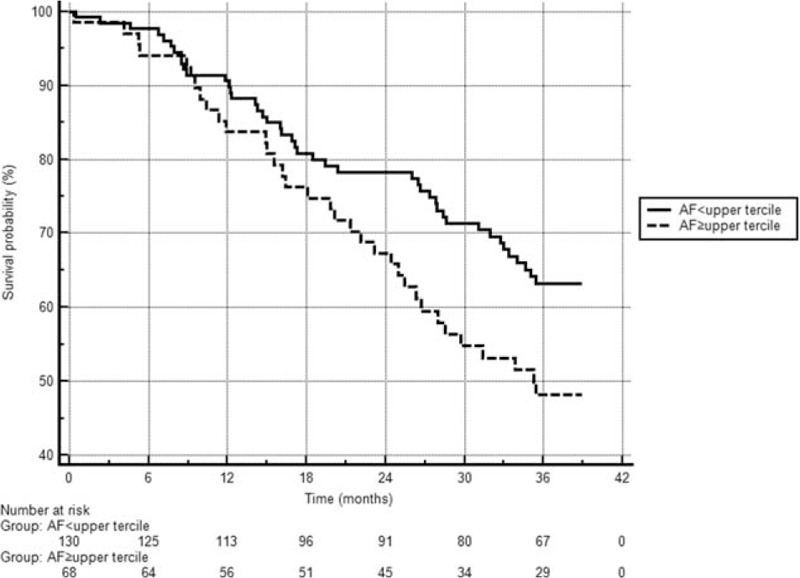
Survival results according to high or low skin autofluorescence values. Patients in the lower SAF (SAF < 3.61) group (n = 130) had better survival results than patients in the higher SAF group (n = 68) (*P* = 0.04). Mortality in the low SAF group was 33.9% (44/130); mean survival was 31.8 months (SE 1.0, 95% CI 29.9–33.8). Mortality in the high SAF group was 50% (34/68); mean survival was 28.6 months (SE 1.47, 95% CI 25.8–31.5). CI = confidence interval, SAF = skin autofluorescence, SE = standard error.

**FIGURE 2:**
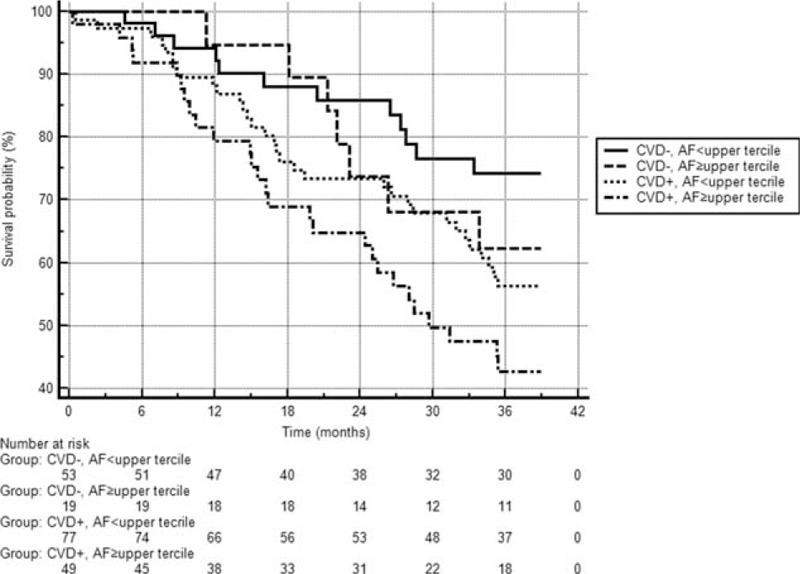
Survival results according to high or low skin autofluorescence values stratified according to the presence of cardiovascular disease. Patients were stratified into the lower (SAF < 3.61) and higher SAF groups with (CVD[+]) or without presence of CVD (CVD[−]). Mortality in the CVD(−)low SAF group was 22.6% (12/53), mean survival was 33.9 months (SE 1.39, 95% CI 31.2–36.6). Mortality in the CVD(−)high SAF group was 36.8% (7/19), mean survival was 32.7 months (SE 2.1, 95% CI 28.7–36.7). Mortality in the CVD(+) low SAF group was 41.6% (32/77), mean survival was 30.5 months (SE 1.37, 95% CI 27.8–33.1). Mortality in the CVD(+) high SAF group was 55.1% (27/49), mean survival was 27.1 months (SE 1.83, 95% CI 23.5–30.6). CI = confidence interval, CVD = cardiovascular disease, SAF = skin autofluorescence, SE = standard error.

We observed the survival of our patients over a 36-month period dividing the patient population into higher and lower SAF groups (according to the upper tercile SAF level 3.61 AU cut-off value). Comparing the survival curves, the lower SAF group had better results (*P* = 0.04) (Fig. 1). Next, the patients were further stratified according to the presence of CVD and SAF levels. The advantage of a lower SAF was shown in both the CVD(+) and CVD(−) status groups (Fig. 2). There were significant differences in survival in cases of CVD(−) with low SAF group compared with the CVD(+) low SAF group (*P* = 0.03), and in the CVD(−) low SAF group versus the CVD(+) high SAF group (*P* < 0.01).

## DISCUSSION

Peritoneal glucose absorption can change body composition, dyslipidemia, and glycemic control, and putatively increase cardiometabolic risk through several pathways.^30^ Cumulative metabolic stress and the repetitive transient hyperglycemia due to PD fluids result in elevated levels of tissue AGE in dialysis patients and ensuing systemic CVD.^31^ Plasma AGE levels have been reported to be significantly higher after a 3-month exposure to high glucose-degradation product (GDP) solution than after treatment with low-GDP fluid, thus worsening the cardiovascular risk profile of dialyzed children.^32^ These observations are in line with our results, indicating that systemic glucose load can contribute to tissue AGE accumulation and vascular changes, and can eventually predict mortality. Measurement of SAF is gaining widespread approval in the diagnosis of diabetes and intermediate carbohydrate tolerance disturbances, and screening values are empowered by the well known performance of SAF in predicting diabetic complications.^33^ Measurement of SAF can indicate tissue alterations long before they would be revealed by conventional screening methods or cause clinical signs.^34^

There is a lack of data from large prospective trials regarding the effects of different PD solutions in patients with and without diabetes on mortality. Therefore, it is difficult to make definite conclusions about the clinical significance of PD-related glucose exposure.^35^ However, a recent evaluation revealed there was an association between SAF and all-cause (2.09-fold increase for each 1 AU increase in AGE values) and sepsis-related mortality in PD patients, albeit the number of patients (n = 102) were limited.^36^ Better survival with biocompatible PD solutions has also been observed in early studies.^37^ PD is associated with an obligatory absorption of carbohydrates, although clinical benefit of glucose-sparing regimens are inconsistent and potentially small,^38^ according to even the most recent analysis. Biocompatible PD solutions, with properties including neutral pH and low GDP regimes, can help to preserve higher residual renal function and urine volume, whereas icodextrin prevents fluid overload by improving peritoneal ultrafiltration in PD.^39^

Some clinicians have offered biocompatible, nonglucose-based PD solutions to diabetic patients for several years. The preference of low-GDP PD solutions and elective introduction of icodextrin are considered to be part of general therapeutic strategies of diabetic patients on PD.^40^ Biocompatible and nonglucose-based PD solutions might have advantageous effect on long-term membrane degeneration, as well as on systemic metabolic control.^41^ Icodextrin has a well known beneficial effect in the management of high transporter diabetic patients on PD by improving peritoneal ultrafiltration and simultaneously reducing overexposure to glucose.^42^

In line with the metabolic memory theory in diabetic patients starting PD, the prevalence of CVD is three times higher than in nondiabetic counterparts, which partially explains the increased morbidity rates and lower survival observed in diabetic patients with CVD.^43^ According to the analysis of predialysis patients with CKD, SAF was found to be independently correlated to renal failure progression. Hence, it seems to be a suitable tool for risk stratification in this population. This relationship was more pronounced in advanced stages of renal failure and presence of diabetes.^44^ In a prospective cohort of CKD stage 3 patients, SAF correlated significantly with a poorer 3.6-year survival, but this association was attenuated to include CVD, diabetes, estimated glomerular filtration rate (eGFR), microalbuminuria, and other established risk factors.^45^ Metastatic calcification might be the putative link to mortality. In patients with stage 3 to stage 5 CKD, multislice computed tomography estimated a coronary artery calcification score (CACS) >400, correlated with SAF, even when adjusting for age, CRP, eGFR, and intact PTH (iPTH).^46^

Improvements in risk of mortality in patients on dialysis will in large part be dependent on the rate of decline in eGFR values during predialysis care.^47^ Better management of diabetic kidney disease (DKD) in the earlier stages can slow down rates of disease progression and premature death from cardiovascular causes. Molecular pathways of peritoneal membrane remodeling are similarly complicated, AGE products represent theoretical mediators, and hence newer PD solutions along with specific inhibitors can help to develop future strategies for preservation of peritoneal membrane and residual kidney function.^48^

We note some limitations to our study. We assessed patients currently undergoing PD; however, we ignored their previous hemodialysis period, and 3 patients returned to PD more than 1 year after failure of their kidney graft. We calculated glucose burden according to the prescription used, which can be a bit different from the amount actually provided, except for 27 APD patients. We should expect a potential presence of additional hidden malignancies (mean ages were 55.8 and 66.2 y, diabetes ratios were 25% and 59.5% in the group without CVD and with CVD, respectively) in the study cohort during the observation period; however, there were only 5 reported deaths. Because of the known adverse effect of jaundice on SAF measurements, we excluded all patients with hepatic disease; thus the results did not exactly reflect the conventional PD population.

In conclusion, SAF measurements in the PD population may help to evaluate the previous cumulative metabolic burden, and SAF values over 3.61 AU provide a reliable cut-off for predicting 3-year mortality.
